# Dysregulated Sheddase Signalling as a Molecular Driver of Plaque Instability Revealed by Integrative Transcriptomics

**DOI:** 10.1111/jcmm.71136

**Published:** 2026-04-25

**Authors:** Alaa G. Alahmadi, Hind A. Alkhatabi, Reem M. Alotibi, Wedad M. Albeshri, Mohammed El‐Mezgueldi, Ammar AL‐Farga, Peter Natesan Pushparaj

**Affiliations:** ^1^ Department of Biological Science College of Science, University of Jeddah Jeddah Saudi Arabia; ^2^ Randall Centre for Cell & Molecular Biophysics King's College London London England UK; ^3^ College of Health and Life Sciences, School of Biosciences, University of Aston Birmingham UK; ^4^ Institute of Genomic Medicine Sciences (IGMS), Faculty of Applied Medical Sciences King Abdulaziz University Jeddah Saudi Arabia

**Keywords:** atherosclerotic plaques, next‐generation knowledge discovery, plaque rupture, plaque stability, sheddase signalling

## Abstract

Atherosclerosis is a major cause of mortality due to chronic and progressive low‐grade inflammation and fibroproliferative remodelling of the intima of arteries. Comprehensive understanding of the interplay between plaque biology and the mechanisms underlying plaque vulnerability and rupture is essential. Here, we aimed to investigate the transcriptomic profiles of stable and unstable atherosclerotic plaques using RNA sequencing data from human carotid atherosclerotic plaque samples based on next‐generation knowledge discovery (NGKD) methods. High‐throughput RNA‐seq data from plaques dissected in stable and unstable regions of four patients were obtained from the Gene Expression Omnibus (GEO) database. GEO RNA‐seq Experiments Interactive Navigator (GREIN) software was used to obtain raw gene‐level counts and filtered metadata for this dataset. The data were further filtered and normalized using Express analyst to derive differentially expressed genes (DEGs) in unstable plaques compared to stable plaques. The DEGs were further analysed using WebGestalt, STRING DB, preranked gene set enrichment analysis (GSEA), and Ingenuity Pathway Analysis (IPA) software. We identified 4792 DEGs in unstable plaques based on a *p*‐value cutoff of < 0.05. NGKD analysis revealed that the sheddase pathway, collagen degradation, activation of matrix metalloproteinases (MMPs), and extracellular matrix (ECM) degradation ranked among the top five upregulated pathways, whereas the inhibition of MMPs and smooth muscle contraction pathways were identified as the most prominent downregulated pathways in unstable plaques. We found that the sheddase pathway was one of the most significantly upregulated canonical pathways in unstable plaques and this finding opens new avenues for potential therapeutic interventions in patients with atherosclerosis.

## 1 Introduction

Atherosclerosis is a chronic and progressive disease that is the leading cause of mortality worldwide. Atherosclerosis is characterized by fibroproliferative remodelling in the intima of medium‐ and large‐sized arteries and low‐grade inflammation [1]. The formation of arterial atherosclerotic plaques drives the clinical manifestations of atherosclerotic cardiovascular diseases (ASCVDs) like myocardial infarction, ischemic stroke, and peripheral artery disease [1]. The latest World Health Organization (WHO) report in 2025 showed that approximately 20 million people died, accounting for approximately 32% of global deaths due to cardiovascular diseases (CVDs).

In 2016, over 45% of all deaths in Saudi Arabia were caused by CVD, and 85% of these deaths were ascribed to heart attacks and strokes. About 201,300 Saudi citizens were affected by CVD in Saudi Arabia in 2016. About 8% of the healthcare budget of Saudi Arabia was allocated to CVDs. By 2035, CVDs were projected to reach $9.8 billion with 480,000 additional cases [2, 3]. Importantly, the CVD cases are predicted to rise by 30%–40%, owing to comorbidities such as diabetes, obesity, hypertension, and unhealthy lifestyles in about 49% of individuals aged 61–75 years and over 30% of adults aged 18 and older [3]. Overall, these statistics highlight the urgent need for preventive strategies and public health interventions to improve CVDs in the Saudi population [3].

**FIGURE 1:**
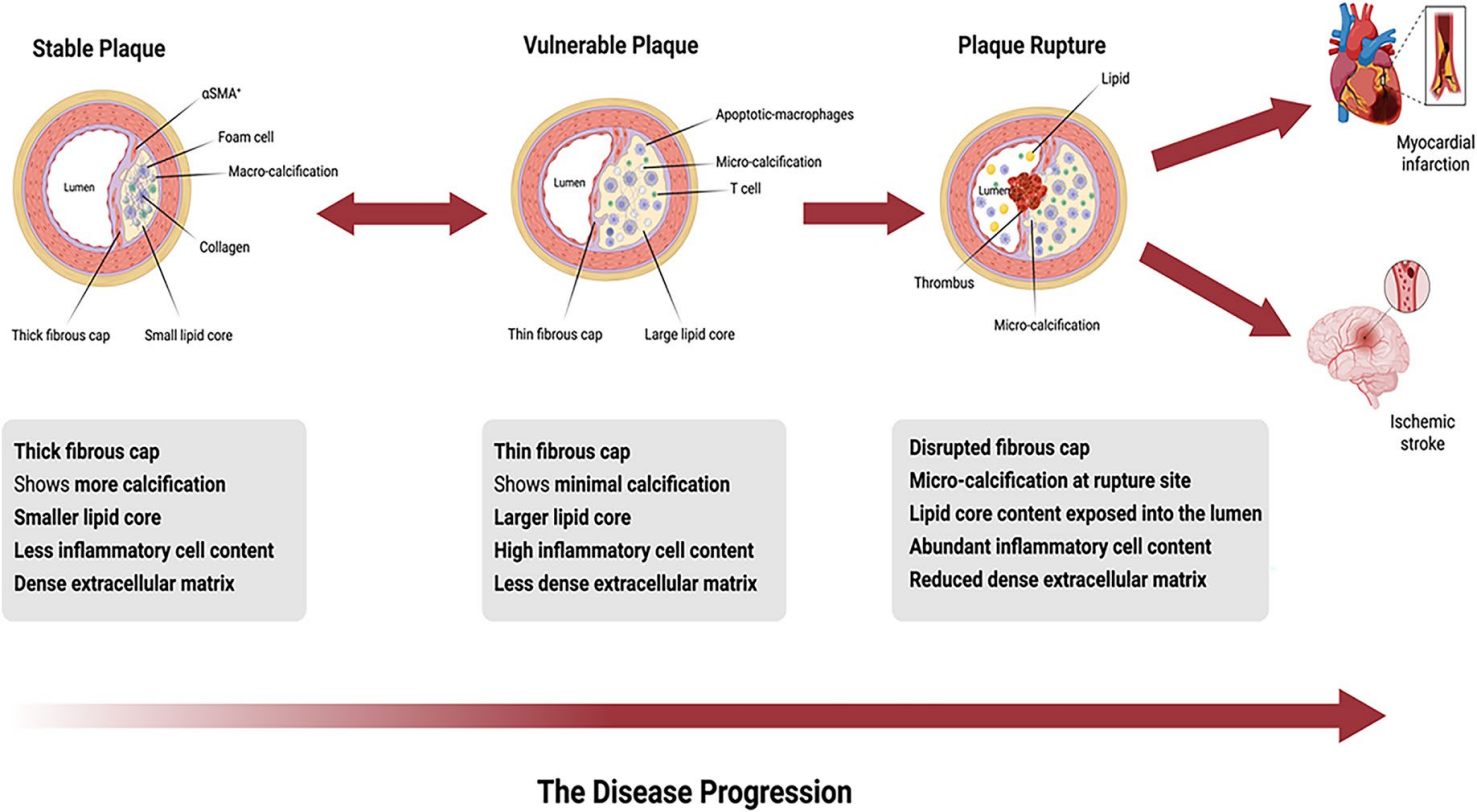
Atherosclerotic Plaque progression: The accumulation of M1 macrophages and T cells reduce the thickness of fibrous cap in stable plaques, exposing the necrotic core and triggering thrombus formation, which can cause myocardial infarction or stroke (Created in https://BioRender.com).

The development of atheromatous plaques is asymptomatic and complex process with a long latency period [4]. The formation of atherosclerotic plaques is initiated by endothelial dysfunction, which promotes the adhesion and infiltration of monocytes and T lymphocytes into the subendothelial intimal layer [5]. Foam cells are formed by the uptake of oxidized LDL‐C by macrophages. The migration and proliferation of vascular smooth muscle cells (VSMCs) from the medial layer into the intima augments the problem and participate in foam cell formation and induce local inflammation [5]. Over time, foam cells undergo apoptosis and necroptosis due to nutrient deprivation in the surrounding medium, intracellular cholesterol accumulation, and oxidative stress [6]. The foam cells are rapidly and efficiently cleared by efferocytosis in the early stages [7]. However, in the late stages of atherosclerotic lesions, Om the contrary, the accumulation of apoptotic foam cells due to defective efferocytosis in the late stages leads to the formation of a necrotic core, pockets of dead macrophages, intracellular debris, and modified lipids [7, 8]. The atherosclerotic lesions then evolve into fibroatheromas, characterized by a necrotic core encapsulated by a fibrous cap primarily composed of smooth muscle cells and extracellular matrix (ECM) proteins, such as elastin and collagen [5] (Figure 1).

Stable plaques are characterized by thick fibrous caps enriched with high levels of α‐smooth muscle actin (αSMA) + and collagen types I and III, along with small lipid cores and determine the structural integrity and stability of plaques [9, 10]. In contrast, unstable plaques with a large lipid‐rich necrotic core covered by a thin inflamed fibrous cap predispose them to rupture, subsequently causing thrombosis [8, 9, 10]. Several cellular and molecular processes collectively lead to the degradation and thinning of the fibrous cap, such as pro‐inflammatory cytokine secretion, heightened secretion of matrix‐degrading enzymes such as matrix metalloproteinases (MMPs), and other proteases that degrade the ECM protein components of the fibrous cap and inhibit the expression of endogenous tissue inhibitors of MMPs (TIMPs) [5]. Importantly, the sheddase signalling pathway has garnered significant attention among the numerous proteolytic mechanisms implicated in this destabilization.

Stable plaques are characterized by thick fibrous caps enriched with high levels of α‐smooth muscle actin (αSMA) + and collagen types I and III, along with small lipid cores. This reduces their susceptibility to rupture and determines the clinical consequences [9, 10]. In contrast, unstable plaques with a large lipid‐rich necrotic core covered by a thin inflamed fibrous cap predispose them to rupture, subsequently causing thrombosis [8, 9, 10]. Several cellular and molecular processes collectively lead to the degradation and thinning of the fibrous cap, such as pro‐inflammatory cytokine secretion, heightened secretion of matrix‐degrading enzymes such as matrix metalloproteinases (MMPs), and other proteases that degrade the ECM protein components of the fibrous cap and inhibit the expression of endogenous tissue inhibitors of MMPs (TIMPs) [5].

In this study, we used Next‐Generation Knowledge Discovery (NGKD) approaches to analyse high‐throughput RNAseq data to decipher the pathological mechanisms associated with the transition from stable to unstable plaques. NGKD methods provide an overall view of differentially regulated canonical pathways and functional protein networks to decode the molecular drivers of plaque stability [11, 12].

Sheddases are membrane proteases that catalyse the cleavage of the extracellular domains of transmembrane proteins. Abnormal sheddase activity in cardiovascular diseases promotes endothelial dysfunction by releasing soluble factors that promote the adhesion and permeability of cells in blood vessels. Moreover, sheddases play a crucial role in plaque destabilization by degrading ECM elements, weakening the fibrous cap, and inducing VSMC apoptosis. The sheddase signalling was further studied in relation to atherosclerotic plaque formation.

Hence, the objectives of this study were to characterize the DEGs of unstable versus stable human carotid atherosclerotic plaques using NGKD methods, identify specific canonical pathways that drive the transition to a pathogenic state, and evaluate the specific role of the sheddase signalling pathway as an upstream regulator of plaque instability to identify novel therapeutic targets to improve vascular stability and prevent acute ischemic events in patients with late‐stage atherosclerosis.

## 2 Materials and Methods

### 2.1 Ethics Statement

This study did not require Institutional Review Board (IRB) approval as it did not involve human participants or animal models, and the data were already de‐identified. We utilized RNA‐sequencing (RNA‐seq) datasets from the Gene Expression Omnibus (GEO) [13]. Sample collection, supplementary processing, and data submission to GEO were conducted with ethical approval from Mahmoud et al. (2019) [14]. This data can be used for further knowledge discovery using NGKD tools, as described previously [12].

### 2.2 Data Source

High‐throughput RNAseq data were obtained from the GEO database (accession number GSE120521). The RNAseq data were obtained from plaques dissected in stable and unstable regions from four patients [14]. As previously described, the GEO RNA‐seq Experiments Interactive Navigator (GREIN) software was used to obtain raw gene‐level counts and filtered metadata for this dataset [11, 12].

### 2.3 High‐Dimensional Data Analysis Using GREIN and Express analyst

GREIN is an open‐source tool designed to reuse RNA‐seq data from GEO [11] and can be accessed at https://shiny.ilincs.org/grein (accessed September 19, 2025). The raw gene‐level data and metadata from the GSE120521 dataset obtained from the GREIN database were filtered and normalized using Express analyst (www.expressanalyst.ca) (accessed September 19, 2025). The normalized data were subsequently used as inputs for the limma‐based differential expression analysis method, which employs a log2‐counts per million (log CPM) transformation and applies filters, such as the low abundance (cutoff value set at 4) and variance filters, as well as a filter for unannotated genes (cutoff value set at 15). The resulting differentially expressed genes (DEGs) were further filtered based on a *p*‐value cutoff of < 0.05 and identified 4792 DEGs in unstable plaques compared with stable plaques, as previously described [12].

### 2.4 WebGestalt Analysis

DEGs were analysed using the WebGestalt tool (wGSEA) [12, 13, 14, 15, 16] using the gene set enrichment analysis (GSEA) method. The differentially regulated pathways were studied using KEGG, Reactome, Wikipathways, and Mitocarta. The Gene Ontology (GO) enrichment analysis for biological processes (GO‐BP), molecular functions (GO‐MF), and cellular components (GO‐CC) affected by unstable plaques was performed. The reference list for each analysis included all mapped gene symbols from the selected platform genome, with the parameters for the enrichment analysis set at a minimum of three and a maximum of 2000 IDs in the category, a false discovery rate (FDR) of *p* < 0.05, computed using the Benjamini–Hochberg (BH) method, and a significance level of the top 10, as described previously [12].

### 2.5 STRING DB And REACTOME Pathway Analyses

DEGs were analysed to investigate the corresponding protein–protein association networks and functional enrichment using STRINGDB v12.0 [17] with an FDR stringency of 1% (high) and *Homo sapiens* as the species. The terms were grouped by similarity ≥ 0.8 and sorted according to the enrichment score. The DEGs obtained based on GSEA analysis from WebGestalt and STRING DB were further analysed using Reactome pathway analysis tools [18].

### 2.6 Ingenuity Pathway Analysis

DEGs were analysed using Ingenuity Pathway Analysis (IPA) software (Qiagen, USA) to explore the differentially regulated canonical pathways in unstable plaques and their relevance in the pathogenesis of atherosclerosis. Briefly, IPA core analysis was used to identify the top 20 canonical pathways that were differentially activated or inhibited in unstable plaques compared with stable (control) plaques using the right‐tailed Fisher's Exact Test and the *Z*‐scores as previously described [19, 20].

### 2.7 Preranked Gene Set Enrichment Analysis

Gene set enrichment analysis (GSEA) against a ranked list of DEGs derived from the comparison of unstable versus stable plaques using the preranked method was performed as previously described [21]. Briefly, the pre‐ranked GSEA analysis was performed by weighting each gene's contribution to the enrichment score according to the value of its ranking metric against gene matrix (GMT) files for Hallmark gene sets v2025.1. Hs (updated June 2025) from the Molecular Signatures Database (MSigDB) using the Java‐based desktop application GSEA 4.3.2 (Broad Institute, USA), with default settings as previously described [12, 13, 14, 15, 16, 17, 18, 19, 20, 21, 22, 23].

## 3 Results

**FIGURE 2:**
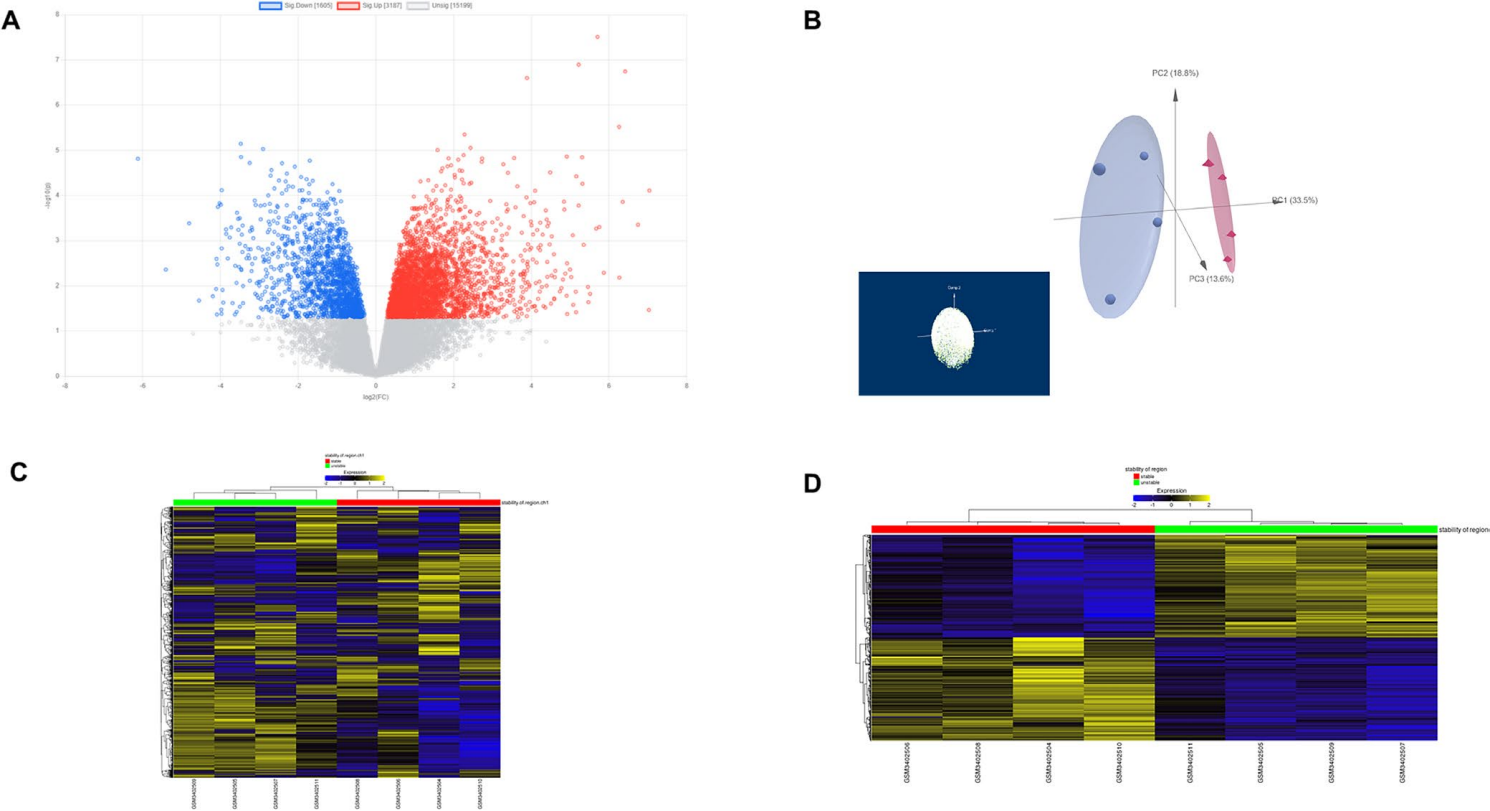
GREIN and Express analyst Analyses. The raw counts and filtered metadata (GSE120521) from GREIN, the DEGs were obtained using Express analyst based on a *p*‐value cutoff of < 0.05, as depicted in the (A) volcano plot, (B) principal component analysis (PCA), (C) heatmap based on the raw counts of the top 5000 genes, and (D) the top 1000 DEGs ranked by adjusted *p*‐values and Pearson correlation.

Here, we performed NGKD analysis of the GEO dataset GSE120521 obtained from RNA‐seq experiments to uncover the DEGs in unstable plaques compared to stable plaques in patients with atherosclerosis. Using the raw counts and filtered metadata from GREIN, we identified 4792 DEGs using Express analyst based on a *p*‐value cutoff of < 0.05, as depicted in the volcano plot (Figure 2A) and the corresponding principal component analysis (PCA) (Figure 2B). The heatmap based on the raw counts of the top 5000 genes (Figure 2C) and the top 1000 DEGs (Figure 2D) ranked by adjusted *p*‐values and Pearson correlation was computed using GREIN.

**FIGURE 3:**
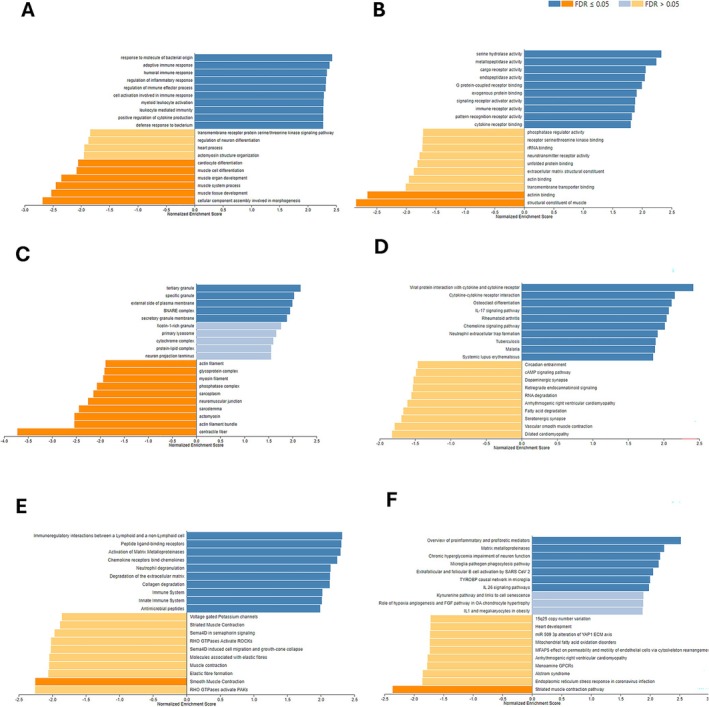
WebGestalt Analyses. (A) Gene Ontology Biological Process (GO‐BP), (B) Gene Ontology‐Molecular Function (GO‐MF), (C) Gene Ontology Cellular Component (GO‐CC), and (D) differentially regulated KEGG, (E) Reactome, and (F) Wikipathways derived from the DEGs obtained from unstable plaques compared to stable plaques.

GO‐BP analysis using the WebGestalt tool showed that leukocyte migration and response to molecules of bacterial origin. The adaptive immune response, regulation of immune effector processes, humoral immune response, leukocyte‐mediated immunity, myeloid leukocyte activation, cell activation involved in immune response regulation of inflammatory response, and positive regulation of cytokine production were positively enriched (FDR < 0.01) in unstable plaques, whereas muscle cell differentiation, muscle system process, muscle organ development, muscle tissue development, and cellular component assembly involved in morphogenesis were negatively (FDR < 0.05) enriched (Figure 3A). GO‐MF analysis based on the WebGestalt tool showed that the serine hydrolase activity, metallopeptidase activity, cargo receptor activity, endopeptidase activity, G protein‐coupled receptor binding.

Exogenous protein binding, signalling receptor activator activity, and immune receptor activity were positively enriched (FDR < 0.05) in unstable plaques; however, transmembrane transporter binding, actinin binding, and structural constituents of muscle were negatively enriched (FDR < 0.05) (Figure 3B).

GO‐CC analysis based on the WebGestalt tool showed that tertiary granule, specific granule external side of plasma membrane, SNARE complex, and secretory granule membrane were positively enriched (FDR < 0.05), whereas myosin complex, myosin filament, actin filament. The phosphatase complex, sarcoplasm, neuromuscular junction, sarcolemma, actin filament bundle, actomyosin, and contractile fibre were negatively enriched (FDR < 0.05) in unstable plaques (Figure 3C).

WebGestalt analysis of DEGs derived from unstable plaques based on KEGG pathways revealed that the genes involved in viral protein interaction with cytokine and cytokine receptor, cytokine‐cytokine receptor interaction, IL‐17 signalling pathway, osteoclast differentiation, chemokine signalling pathway, neutrophil extracellular trap (NET) formation, and NF‐kappa B signalling pathway were significantly enriched (FDR < 0.05), whereas peptide vascular smooth muscle contraction (Figure 3D) was negatively enriched in unstable plaques compared to stable plaques.

WebGestalt analysis of DEGs derived from unstable plaques based on the Reactome database revealed that the genes involved in immunoregulatory interactions between a lymphoid and a non‐lymphoid cell, Activation of matrix metalloproteinases, Peptide ligand‐binding receptors, chemokine receptors bind chemokines, neutrophil degranulation, degradation of the extracellular matrix, collagen degradation, Innate immune system, Immune system, and Antimicrobial peptides were significantly enriched (FDR < 0.05), whereas striated muscle Contraction, Laminin interactions, Sema4D in semaphorin signalling, RHO GTPases activate ROCKs, Sema4D induced cell migration and growth‐cone collapse, elastic fibre formation, molecules associated with elastic fibres, and muscle contraction. RHO GTPases activate PAKs and Smooth muscle contraction was negatively enriched (*p* < 0.05) in unstable plaques (Figure 3E).

WebGestalt analysis of DEGs derived from unstable plaques based on Wikipathways revealed that the genes involved in Overview of proinflammatory and profibrotic mediators, Matrix metalloproteinases, Chronic hyperglycemia impairment of neuron function, Microglia pathogen phagocytosis pathway, Extrafollicular and follicular B cell activation by SARS CoV 2, TYROBP causal network in microglia, IL 26 signalling pathways, IL1 and megakaryocytes in obesity, Kynurenine pathway and links to cell senescence, and Role of hypoxia angiogenesis and FGF pathway in OA chondrocyte hypertrophy were enriched significantly (FDR < 0.05), whereas the genes involved in miR 509 3p alteration of the YAP1 ECM axis, arrhythmogenic right ventricular cardiomyopathy, and mitochondrial fatty acid oxidation disorders.

Heart development, 15q25 copy number variation, MFAP5 effect on permeability and motility of endothelial cells via cytoskeleton rearrangement, Alstrom syndrome, endoplasmic reticulum stress response in coronavirus infection, sudden infant death syndrome susceptibility pathways, and striated muscle contraction pathway were negatively enriched (*p* < 0.05) in unstable plaques (Figure 3F).

**FIGURE 4:**
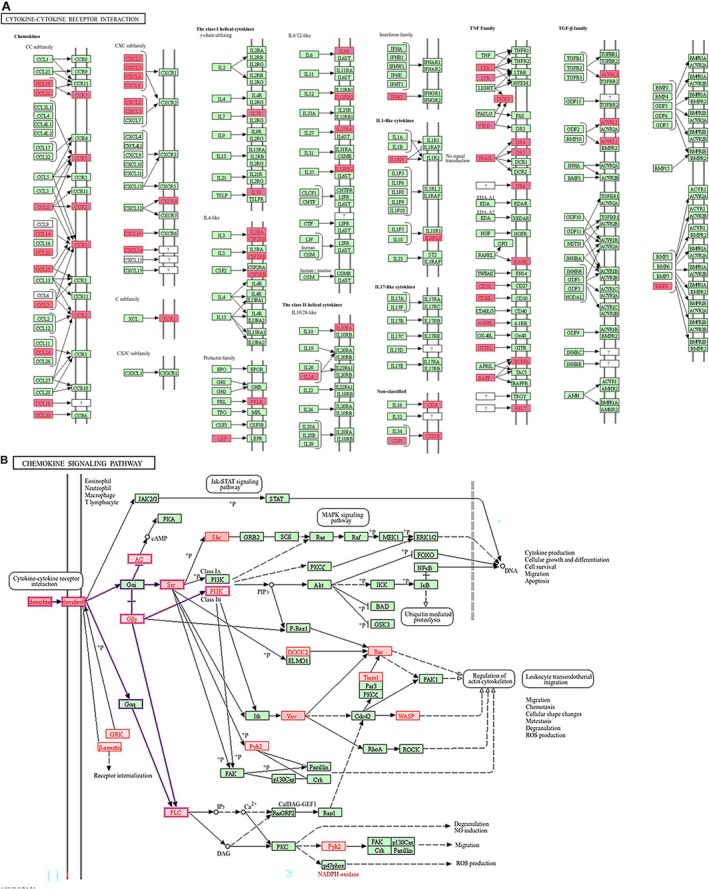
Differentially regulated genes (highlighted in red) in (A) Cytokine‐Cytokine Receptor Interactions and (B) Chemokine Signalling based on KEGG pathways in unstable plaques of atherosclerosis patients.

**FIGURE 5:**
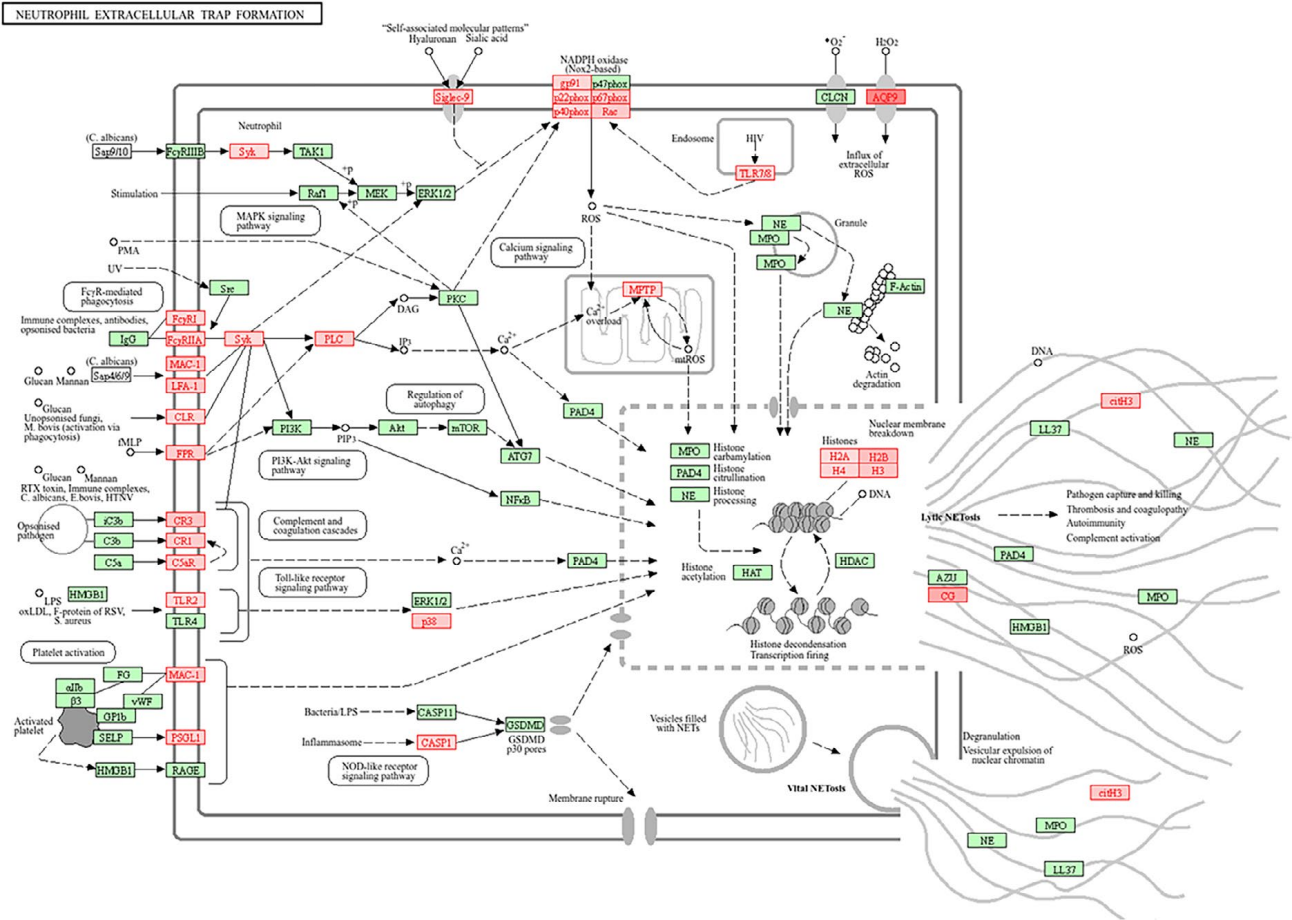
Neutrophil Extracellular Trap (NET) Formation (KEGG). The DEGs upregulated (*highlighted in red*) in NET formation in unstable atherosclerotic plaques.

**FIGURE 6:**
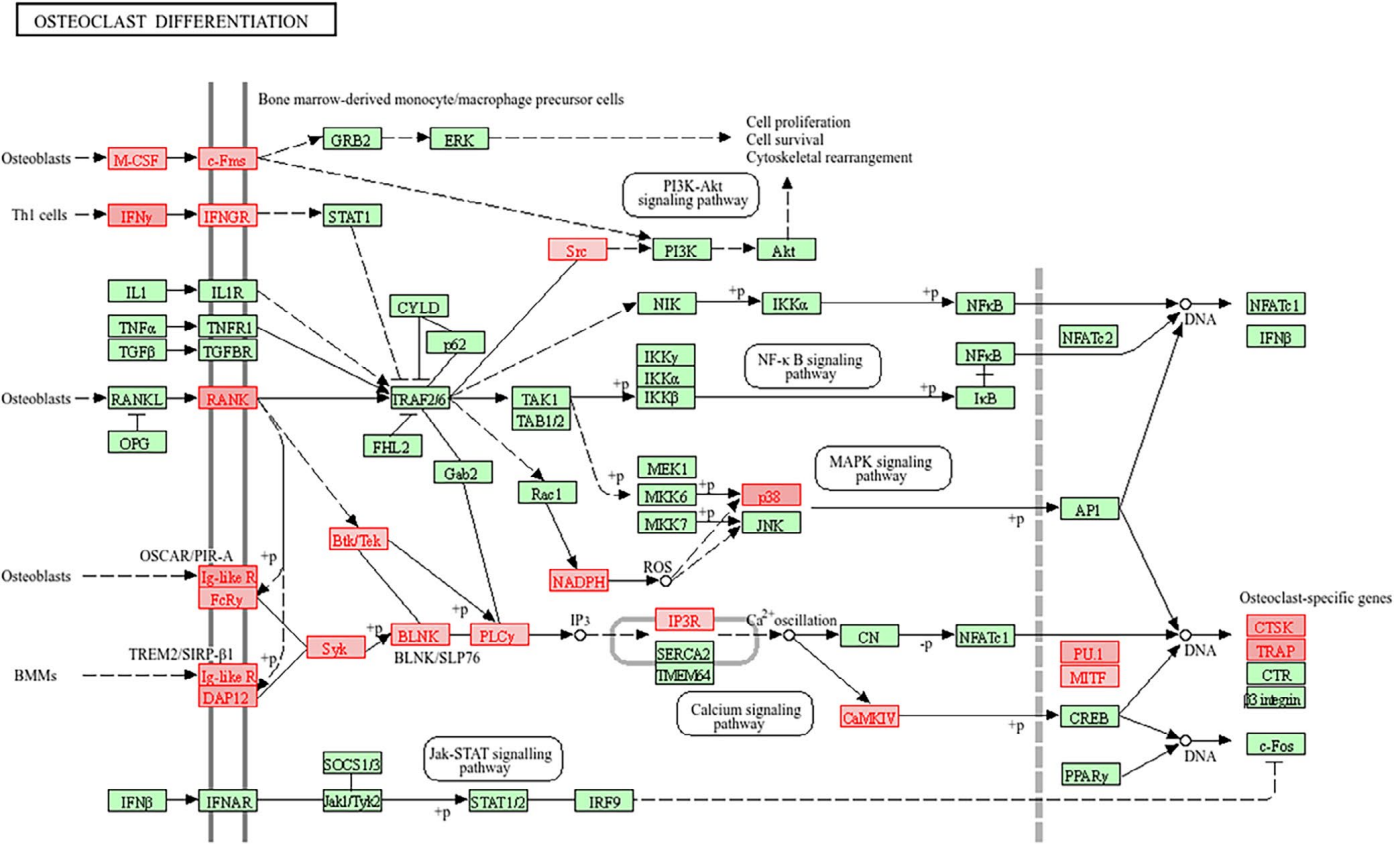
Osteoclast Differentiation (KEGG). The DEGs upregulated (highlighted in red) in osteoclast differentiation in the unstable plaque of patients with atherosclerosis.

More importantly, the WebGestalt analysis based on KEGG pathways showed that most of the genes implicated in Cytokine‐Cytokine Receptor (Figure 4A) and Chemokine Signalling pathways (Figure 4B) were significantly upregulated in unstable plaques. In addition, genes involved in neutrophil extracellular trap formation (Figure 5) and osteoclast differentiation (Figure 6) were significantly upregulated in unstable plaques of atherosclerosis patients (File S1).

**FIGURE 7:**
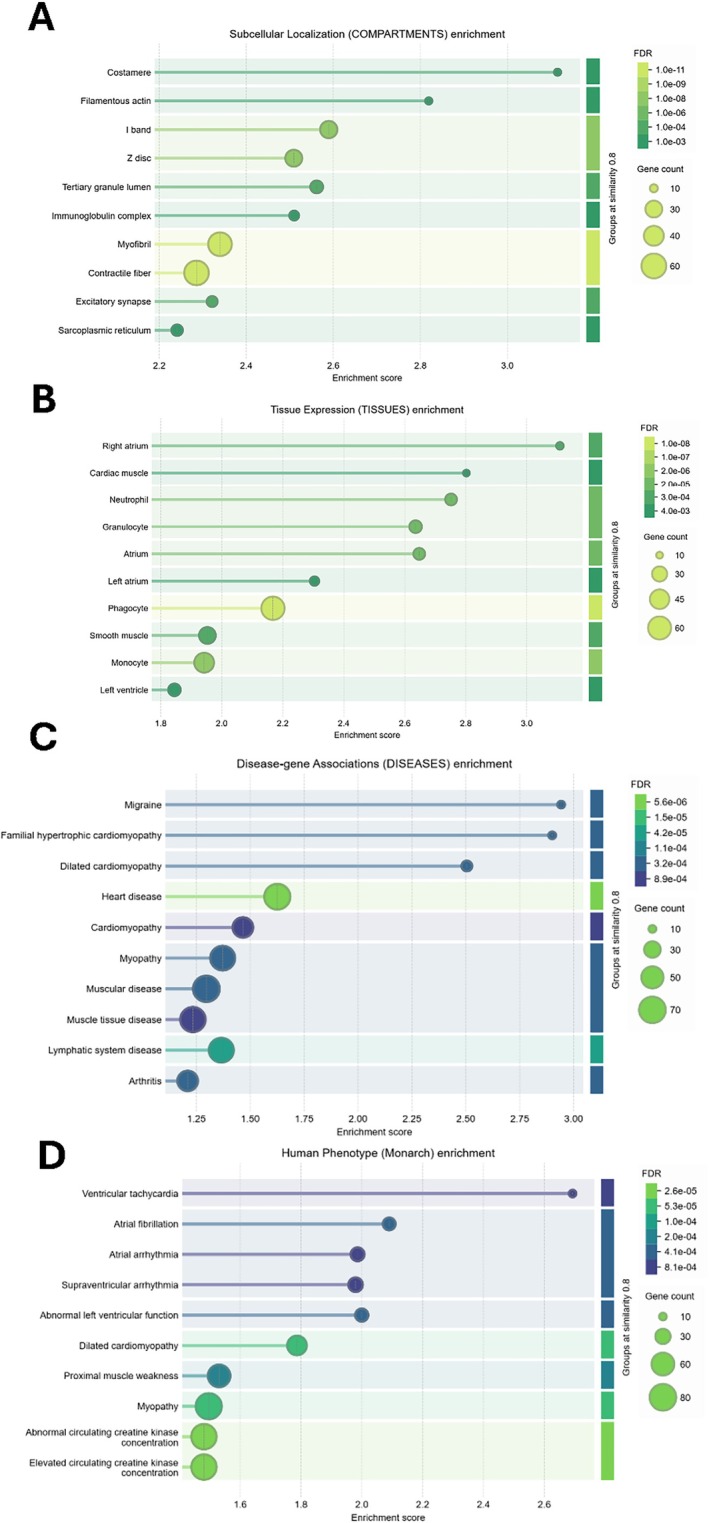
STRING DB Analysis. The corresponding proteins derived from the DEGs enriched in (A) subcellular localization (compartments), (B) Tissue Expression (tissues), (C) Disease‐gene Associations (diseases), and (D) Human Phenotype (monarch) in the unstable plaques of atherosclerosis patients.

Based on STRING DB analysis, the proteins expressed in various subcellular compartments, such as myofibrils, contractile fibres, sarcomeres, I bands, Z discs, focal adhesions, cell‐substrate junctions, Sarcolemma, Actin cytoskeleton, cation channel complex, basement membrane, Actomyosin, Costamere, Actin filament, and sarcoplasm, were negatively enriched (FDR < 0.05) in unstable plaques (Figure 7A). The proteins expressed in tissues such as the sarcoplasmic reticulum in the Atrium, Right atrium, smooth muscle, cardiac muscle, Artery, Temporal lobe, left atrium, skeletal muscle, left ventricle, heart ventricle, and aorta were negatively enriched (FDR < 0.05) in unstable plaques (Figure 7B).

Furthermore, the proteins implicated in Heart disease, Dilated cardiomyopathy, Migraine, Muscular disease, Familial hypertrophic cardiomyopathy, Cardiovascular system disease, Myopathy, Cardiomyopathy, and Muscle tissue disease were differentially regulated (FDR < 0.05) in unstable plaques (Figure 7C). The proteins implicated in Dilated cardiomyopathy, Myopathy, Electrocardiography, PR interval, Abnormality of the musculature of the limbs, Proximal muscle weakness, EMG abnormality, Skeletal muscle atrophy, Abnormal myocardium morphology, Abnormal left ventricular function, Atrial fibrillation, Supraventricular arrhythmia, Cardiomyopathy, Atrial arrhythmia, and Ventricular tachycardia based on Human Phenotype (Monarch) were differentially regulated in unstable plaques (Figure 7D).

**FIGURE 8:**
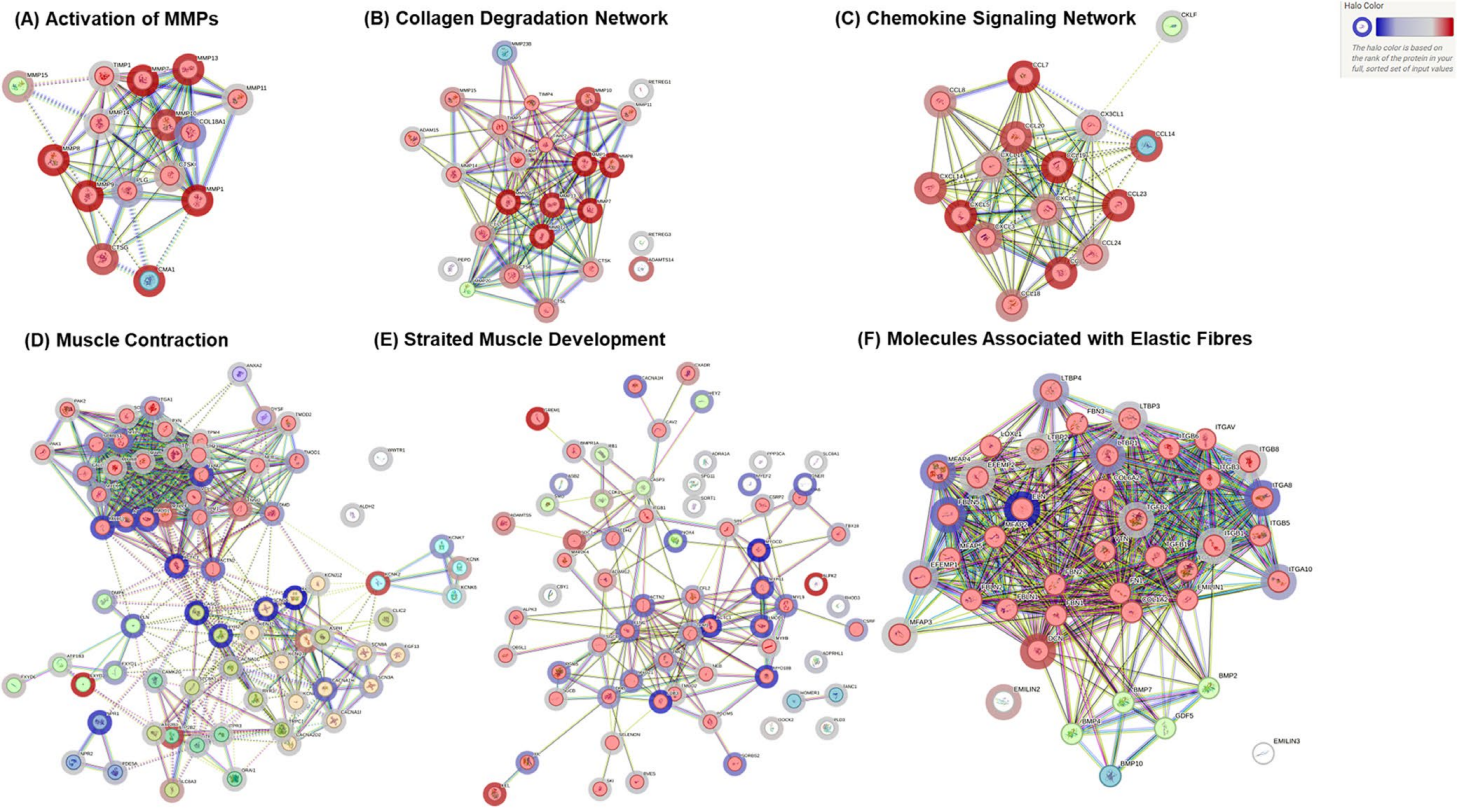
Network Analysis using STRING DB. Some of the key positive enrichment of networks such as (A) Activation of MMPs, (B) Collagen Degradation, (C) Chemokine Signalling and negative enrichments such as (D) Muscle Contraction, (E) Striated Muscle Development, and (F) Molecules Associated with Elastic Fibres in the unstable plaque.

We performed K‐means cluster analysis of the proteins using STRING DB to show the positive enrichment of the Activation of MMPs, Collagen Degradation, and Chemokine Signalling and the negative enrichment of protein networks associated with muscle contraction, striated muscle development, and molecules associated with elastic fibres in the unstable plaques of atherosclerosis patients (Figure 8).

**FIGURE 9:**
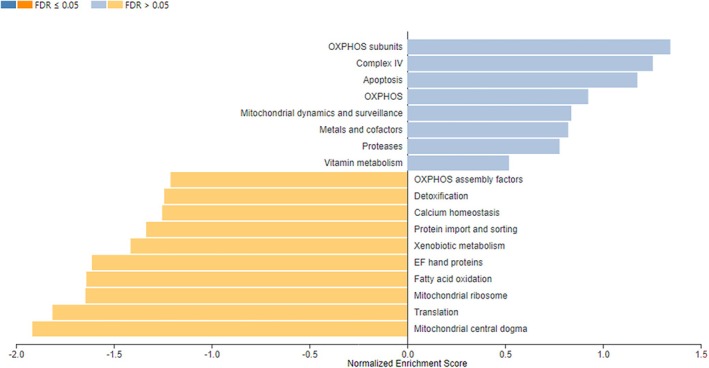
Mitocarta pathway analysis of the DEGs derived from unstable plaques compared to stable plaques using WebGestalt.

The analysis of DEGs using Mitocarta in the WebGestalt tool showed that genes implicated in mitochondrial ribosomes, fatty acid oxidation, translation, and mitochondrial central dogma were negatively enriched (*p* < 0.05) in unstable plaques (Figure 9).

**FIGURE 10:**
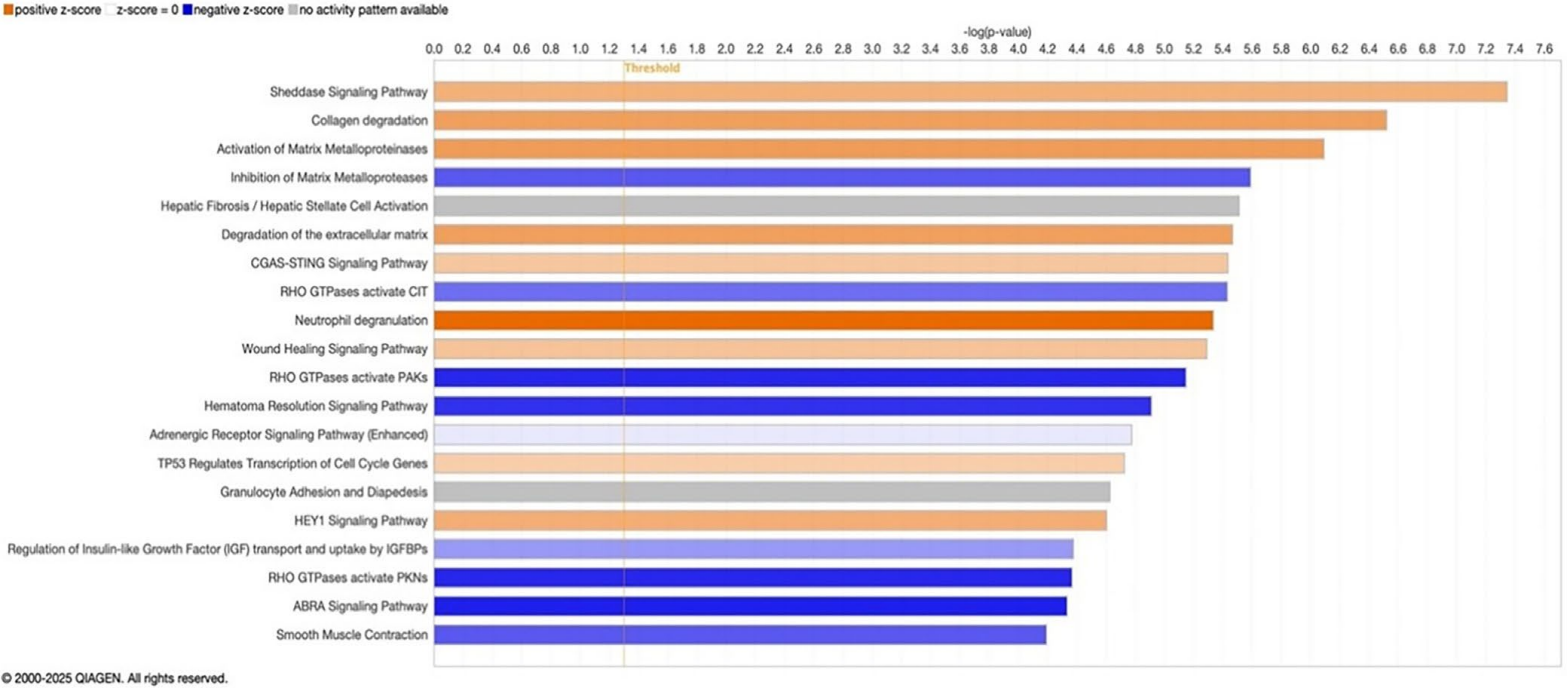
The top 20 differentially regulated canonical pathways implicated in atherosclerosis pathogenesis based on IPA analysis. The canonical pathways are shown on the y‐axis, and the x‐axis represents the −log(*p*‐value) derived from the right‐tailed Fisher's Exact Test. Blue and orange bars indicates downregulation and upregulation, respectively, based on their Z‐scores. The grey bars represent pathways with no activity, and the white bars indicate pathways with a z‐score close to zero. The thin orange vertical line corresponds to the ratio which was calculated based on the number of genes meeting the inclusion criteria divided by the total number of genes involved in that pathway (IPA, Qiagen, USA).

IPA software was used to identify the canonical pathways associated with transcripts that were differentially activated or inhibited in unstable plaques compared with stable (control) plaques with adjusted *p* < 0.05. The analysis revealed that the sheddase pathway, collagen degradation, activation of matrix metalloproteinases (MMPs), and extracellular matrix (ECM) degradation ranked among the top five upregulated pathways, whereas the inhibition of MMPs and smooth muscle contraction pathways was identified as the most prominent downregulated pathways, ranking first and ninth, respectively (Figure 10).

**FIGURE 11:**
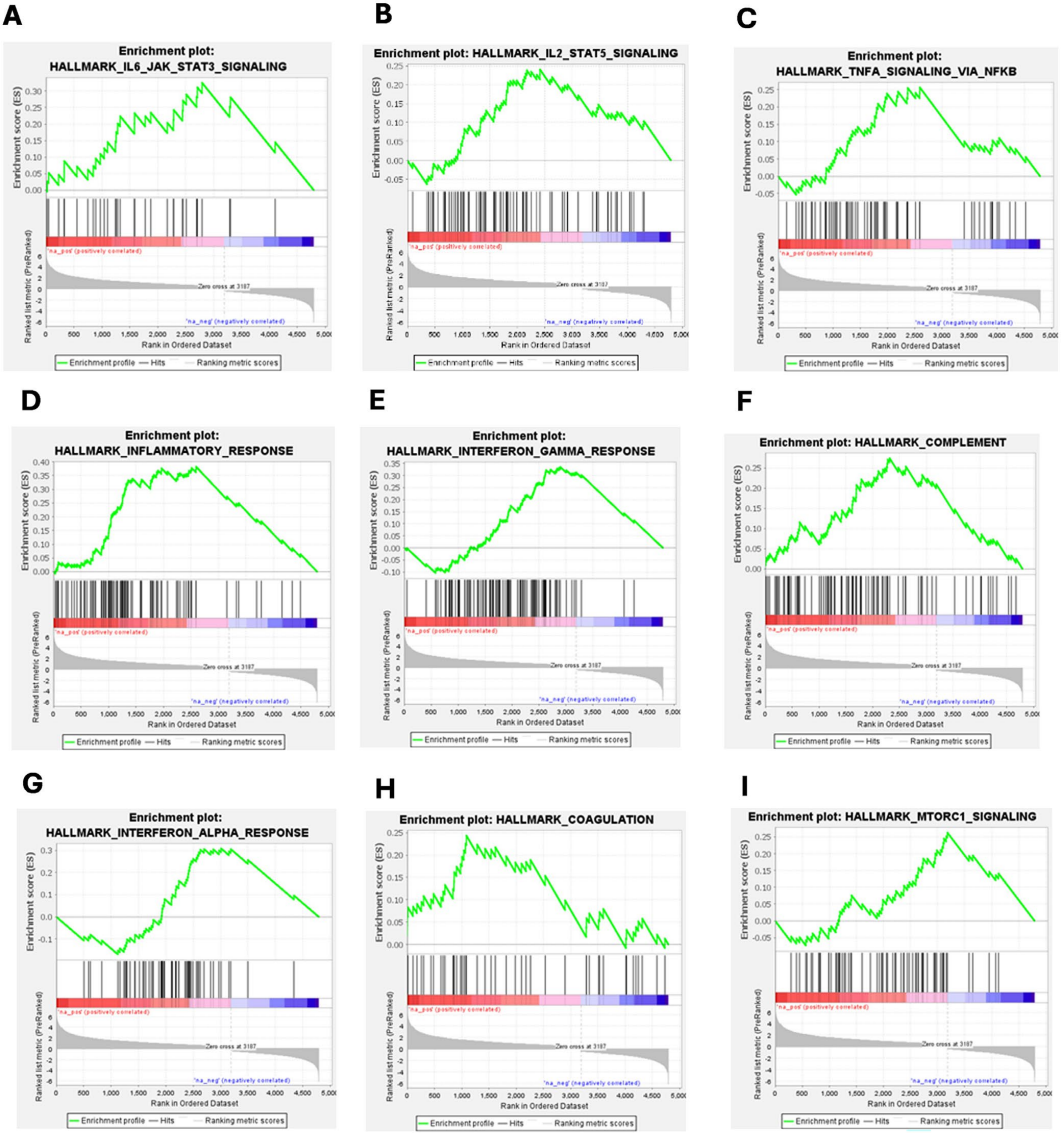
Preranked Gene Set Enrichment Analysis (GSEA) was performed using GSEA software (version 4.4; Broad Institute, USA) for the Hallmark gene sets. Positive enrichment of (A) Inflammatory Response, (B) Interferon Gamma Response, (C) Complement, (D) Interferon alpha Response, (E) IL‐2 STAT 5 Signalling, (F) TNF alpha Signalling_Via_Nfkb, (G) IL‐6 Jak Stat3 Signalling, (H) MTORC1 Signalling, and (I) Coagulation were observed in unstable plaques.

The preranked GSEA analysis of the DEGs showed the positive enrichment of Hallmark Gene Sets such as Inflammatory Response, Interferon Gamma Response, Complement, Interferon alpha Response, IL‐2 STAT 5 Signalling, TNF alpha Signalling_Via_Nfkb, IL‐6 Jak Stat3 Signalling, MTORC1 Signalling, and Coagulation (Figure 11) and the preranked GSEA metrics of all the Hallmark gene sets are shown in Table 1.

**TABLE 1:** The Pre‐Ranked GSEA Metrics for Hallmark Gene Sets based on GSEA 4.4.

S.No	Gene Set	Size	Enrichment Score (ES)	Normalized ES	Nominal *p*‐value	FDR *q*‐value	FWER *p*‐value
1	Hallmark_inflammatory_response	95	0.38	4.4	0	0	0
2	Hallmark_allograft_rejection	92	0.35	4.05	0	0	0
3	Hallmark_interferon_gamma_response	103	0.34	4.04	0	0	0
4	Hallmark_kras_signalling_up	91	0.33	3.82	0	0	0
5	hallmark_complement	93	0.27	3.07	0	0	0
6	Hallmark_e2f_targets	74	0.29	2.97	0	0	0
7	Hallmark_interferon_alpha_response	59	0.31	2.8	0	0	0
8	Hallmark_g2m_checkpoint	81	0.25	2.65	0	0	0
9	Hallmark_il2_stat5_signalling	77	0.24	2.46	0	0.001	0.008
10	Hallmark_mtorc1_signalling	65	0.26	2.46	0	0.001	0.008
11	Hallmark_tnfa_signalling_via_nfkb	63	0.26	2.43	0	0.001	0.01
12	Hallmark_il6_jak_stat3_signalling	32	0.33	2.14	0.002	0.006	0.048
13	Hallmark_myc_targets_v1	31	0.31	2.02	0.004	0.01	0.088
14	Hallmark_coagulation	47	0.24	1.97	0.006	0.013	0.129
15	Hallmark_p53_pathway	68	0.18	1.72	0.022	0.048	0.422
16	Hallmark_estrogen_response_late	57	0.18	1.62	0.045	0.076	0.604
17	Hallmark_fatty_acid_metabolism	41	0.2	1.5	0.062	0.125	0.801
18	Hallmark_reactive_oxygen_species_pathway	16	0.31	1.49	0.065	0.12	0.808
19	Hallmark_apoptosis	66	0.15	1.43	0.107	0.15	0.885
20	Hallmark_glycolysis	68	0.15	1.38	0.114	0.173	0.926
21	Hallmark_peroxisome	20	0.23	1.25	0.209	0.282	0.989
22	Hallmark_cholesterol_homeostasis	29	0.19	1.23	0.214	0.289	0.992
23	Hallmark_uv_response_up	49	0.15	1.22	0.219	0.287	0.992
24	Hallmark_spermatogenesis	30	0.19	1.21	0.222	0.282	0.993
25	Hallmark_angiogenesis	17	0.23	1.15	0.292	0.333	0.999
26	Hallmark_heme_metabolism	50	0.13	1.07	0.355	0.41	1
27	Hallmark_pi3k_akt_mtor_signalling	30	0.16	1.03	0.38	0.443	1
28	Hallmark_androgen_response	35	0.13	0.95	0.492	0.538	1
29	Hallmark_hypoxia	68	0.1	0.94	0.53	0.531	1
30	Hallmark_xenobiotic_metabolism	68	0.09	0.9	0.568	0.573	1

## 4 Discussion

Our study provides a comprehensive NGKD analysis of the molecular mechanisms associated with atherosclerotic plaque instability. We selected the GEO dataset GSE120521 since it has high‐throughput RNAseq data derived from human carotid atherosclerotic plaques [14]. The plaques obtained from the same patient were further dissected into stable and unstable regions and it reduces the inter‐individual genetic variation. We identified that the sheddase pathway was the top canonical pathway upregulated in unstable plaques.

Sheddases are a group of membrane proteases that catalyse the cleavage of substrates and serve as crucial molecular switches that regulate a wide range of cellular and molecular processes [24, 25]. The inflammatory signalling pathways, such as cytokine–cytokine receptor interactions, chemokine signalling, and NF‐κB activation, are well‐known drivers of plaque inflammation, which were differentially regulated; the sheddase pathway has not been extensively studied or reported in the context of atherosclerosis. NGKD analyses further showed increased extracellular matrix degradation, inflammation, immune activation, and reduced structural maintenance and smooth muscle integrity in unstable plaques. It was shown that the cytokines, growth factors, and adhesion molecules are released due to aberrant sheddase activity and contribute to endothelial dysfunction by enhancing leukocyte adhesion and migration and increasing vascular permeability in atherosclerosis [26, 27]. Aberrant sheddase signalling contributes to the degradation of ECM components and weakening of the structural integrity of the fibrous cap, triggering VSMC apoptosis and reducing collagen synthesis, contributing to plaque instability in the late stages of atherosclerosis [28, 29]. The vasoactive mediators angiotensin‐converting enzyme (ACE) and endothelin precursors are increased and subsequently impair VSMC contractility and vasomotor responses due to sheddases [30].

The biological processes associated with immune responses, including leukocyte migration and adaptive immune responses, within unstable plaques proves that inflammation plays a critical role in plaque destabilization [31]. Conversely, pathways associated with muscle cell differentiation and development were downregulated, indicating a phenotypic shift in plaque composition.

Moreover, pathway enrichment analyses based on KEGG, Reactome, and Wikipathways indicated that the cytokine‐cytokine receptor interaction, chemokine signalling, IL‐17 signalling, and NF‐κB pathways were significantly upregulated in unstable plaques contributing to plaque instability and rupture [32, 33].

The decreased mitochondrial fatty acid oxidation suggests that the unstable plaques undergo metabolic dysfunction and are deficient in cellular energy metabolism. NET activation signals a pro‐inflammatory milieu wherein neutrophils release chromatin fibres in addition to proteolytic enzymes, such as proteases and myeloperoxidase, driving local inflammation, weakening the protective endothelial barrier, and promoting plaque erosion and thrombosis [34, 35]. An upregulation of the osteoclast differentiation pathway suggests that macrophages, neutrophils, and osteoclasts can become more active and contribute to calcium deposition as well as matrix destruction, leading to the fibrous cap structural weakening in atherosclerotic plaques. It raises the probability of plaque rupture, an essential process in most acute coronary events including myocardial infarction or stroke [36, 37, 38]. Conversely, in unstable plaques, pathways related to vascular smooth muscle contraction, elastic fibre synthesis, and mitochondrial fatty acid oxidation were reduced to considerable extents, key factors in atherosclerosis and thoracic aortic aneurysms [39, 40], and induce a systemic shift from vascular repair and stability to inflammation and degradation, the hallmarks of plaque instability and vulnerability [41].

The IPA analysis revealed that the sheddase pathway was the top‐ranking canonical pathway activated in unstable plaques followed by collagen degradation and MMP activation. Besides, the MMPs inhibition and smooth muscle contraction were the top downregulated pathways based on Z‐scores, with the sheddase signalling pathway being a key upstream molecular switch that connects inflammatory signalling to ECM breakdown in unstable plaques. Though the inflammatory signalling pathways such as cytokine–cytokine receptor interactions and NF‐κB activation are known to be drivers of plaque inflammation, the biological importance of the sheddase pathway was evident in the present study.

By identifying the sheddase pathway as the key driver of plaque susceptibility, its targeted inhibition represents a formidable approach to the treatment of atherosclerosis. Directing this regulatory pathway are small‐molecule inhibitors and monoclonal antibodies against a‐disintegrin and metalloproteinases (e.g., ADAM10 and ADAM17) that our NGKD was able to highlight as important regulators upstream of this pathway. As broad‐spectrum metalloproteinases have previously encountered problems because of off‐target musculoskeletal side effects, recent work on ‘selective shedding inhibition’ aims to selectively target the cysteine‐rich or disintegrin domains of these proteases to achieve isoform specificity. Apart from direct enzyme inhibition, the second‐line strategy to inhibit downstream mediators of sheddase‐mediated activation, such as soluble TNF‐α, IL‐6 receptor (sIL‐6R) and a range of chemokine ligands, should also be addressed. For example, preventing the trans‐signalling process of IL‐6 that is sparked by the sheddase‐mediated release of sIL‐6R might target the pro‐inflammatory effects of this cytokine in vascular wall but not the systemic classical signalling. Moreover, such molecular interventions as antisense oligonucleotides (ASOs) or RNA interference (RNAi) could be exploited to suppress expression of sheddase in plaque microenvironment in order to decrease the “molecular switching” upon ECM degradation and apoptosis of VSMC. By stabilizing the fibrous cap and sustaining VSMC contractility through these targeted therapeutic strategies, the unstable plaques might transition to a more stable phenotype and thus much greater cardiovascular benefit in late‐stage patients may be realized.

## 5 Future Directions

Western blotting, ELISA, and immunohistochemistry methods are required to confirm that the mRNA expression levels of identified DEGs involved in the sheddase pathway and MMP cascades correlate with increased protein abundance and enzymatic activity in human plaque tissues. In vitro studies using primary human VSMCs are needed to investigate how aberrant sheddase‐mediated signalling triggers the phenotypic shift from a contractile to a synthetic or apoptotic state. Besides, single‐cell RNA sequencing (scRNA‐seq) will be vital to decode which specific cell populations are the primary sources of sheddase activity within the unstable plaque microenvironment.

Importantly, the development of isoform‐selective inhibitors for ADAM10 and ADAM17 could prevent the cleavage of pro‐inflammatory substrates without the off‐target effects associated with broad‐spectrum MMP inhibitors. In vitro models should be developed to probe for potential therapeutic effects of inhibiting NET formation and the osteoclast differentiation pathway for localized inflammation and restitution of the calcification–resorption balance in the fibrous cap. Investigating the restoration of mitochondrial fatty acid oxidation, which was significantly downregulated in our analysis, could provide an innovative approach to stabilize plaque bioenergetics and prevent necrotic core expansion. Future research also should assess the ability of the soluble ectodomains released in plaque from sheddase activity to be detected in serum as diagnostic biomarkers for predicting imminent plaque rupture or “vulnerable patients.” The recognized pathways could be a target for molecular imaging probes (e. g, PET/CT, MRI) to non‐invasively detect active sheddase signalling or MMP activation in patients with high‐risk carotid stenosis.

Hence, our comprehensive NGKD approach revealed the key molecular events responsible for atherosclerotic plaque instability and identified the sheddase signalling pathway as a key upstream regulator of plaque vulnerability, acting as a molecular switch linking inflammatory signalling to ECM breakdown. This opens avenues for potential therapeutic interventions aimed at stabilizing plaques and improving cardiovascular outcomes in patients with atherosclerosis.

## Author Contributions

R.M.A., W.M.A. and M.E. participated in the research design, performed NGKD experiments, and contributed to data analysis, writing and editing of the manuscript. A.A. participated in the research design, project administration and supervision. A.G.A., H.A.A. and P.N.P. conceived and designed the experiments, analysed the data, wrote the manuscript, and were responsible for funding and supervision.

## Funding

This work was supported by the University of Jeddah, UJ‐24‐SUTU‐2759.

## Conflicts of Interest

The authors declare no conflicts of interest.

## Supporting information


**Supplementary File 1** Description The supplementary file contains in separate sheets the entire list of 4792 differentially expressed genes (DEGs) identified using Express analyst, Gene Ontology (GO) enrichment results for Biological Process (GO‐BP), Molecular Function (GO‐MF), and Cellular Component (GO‐CC) by WebGestalt, Statistically significant pathways identified across three major databases(KEGG, Reactome, and WikiPathways), DEGs implicated in key identified pathways, including Neutrophil Extracellular Trap (NET) formation, Chemokine Signalling, Cytokine‐Cytokine Receptor Interaction, and Osteoclast Differentiation, Results of the protein–protein interaction (PPI) network enrichment based on STRING DB analysis, and Targeted enrichment analysis of mitochondrial‐localized proteins based on Mitocarta.

## Data Availability

The data that support the findings of this study are available on request from the corresponding author. The data are not publicly available due to privacy or ethical restrictions.
